# Synthesis and crystal structure of a bench-stable pyridinium ketene hemiaminal: 1-(1-eth­oxy­ethen­yl)-2-[meth­yl(phen­yl)amino]­pyridin-1-ium tri­fluoro­methane­sulfonate

**DOI:** 10.1107/S2056989023005741

**Published:** 2023-07-07

**Authors:** Zoe A. Krevlin, Isabella C. Bote, Maria Christina F. Crespo, Christie C. Lam, Colin D. McMillen, Max M. Majireck

**Affiliations:** a Chemistry Department, Hamilton College, 198 College Hill Rd., Clinton, NY 13323, USA; b Department of Chemistry, Clemson University, H.L. Hunter Laboratories, Clemson, SC 29634, USA; Purdue University, USA

**Keywords:** crystal structure, ketene hemiaminal, *N*-quaternized ketene *N*,*O*-acetal

## Abstract

The novel bench-stable *N*-quaternized ketene *N*,*O*-acetal, C_16_H_19_N_2_O^+^·CF_3_O_3_S^−^, was synthesized and its structure determined. The title compound is a rare example of a pyridinium ketene hemiaminal for which a crystal structure has been determined, joining the 2-chloro-1-(1-ethyoxyethen­yl)pyridin-1-ium tri­fluoro­methane­sulfonate salt from which it was synthesized. The cationic species of the title compound can be defined by three individually planar fragments assembling into a non-coplanar cation. The phenyl substituent extending from the amino nitro­gen atom and the ethyoxyvinyl substituent extending from the pyridine N atom are oriented on the same side of the mol­ecule and maintain the closest coplanar relationship of the three fragments. Supra­molecular inter­actions are dominated by C—H⋯O inter­actions from the cation to the SO_3_ side of the tri­fluoro­methane­sulfonate anion, forming a two-dimensional substructure.

## Abstract 2

The *N*-quaternized ketene *N*,*O*-acetal, 1-(1-eth­oxy­vin­yl)-2-(meth­yl(phen­yl)amino)­pyridin-1-ium tri­fluoro­methane­sulfonate was synthesized and its structure determined, making it a rare example of this class of compounds to be structurally characterized.

## 1. Chemical context

**Figure 1:**
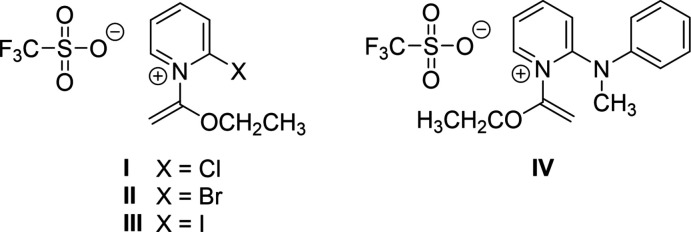
Examples of bench-stable pyridinium ketene hemiaminals (Shapiro *et al.*, 2018 ▸).

*N*-Quaternized ketene *N*,*O*-acetals are a generally unstable class of compounds, most often invoked as reactive inter­mediates (Kantlehner, 2006 ▸). Consequently, there are very few reports of isolable and well-characterized compounds in this class despite their first appearance in the literature over eight decades ago (Arens *et al.*, 1955 ▸; Barnes *et al.*, 1940 ▸; Filippova *et al.*, 1983 ▸; Herkes & Simmons, 1973 ▸; Klages & Drerup, 1941 ▸; Lehn & Seher, 1966 ▸; Otsuru *et al.*, 1969 ▸). In 2018, our laboratory discovered that several pyridinium ketene hemiaminals were unusually stable analogues of the *N*-quaternized ketene *N*,*O*-acetal class, amenable to isolation and purification by chromatography or recrystallization (Fig. 1 ▸, compounds **I**–**III**) (Shapiro *et al.*, 2018 ▸). An ensuing report expanded access to over forty bench-stable examples of this rare class of compounds (McConnell *et al.*, 2021 ▸). However, to date there has been only one published X-ray crystal structure (Fig. 1 ▸, compound **I**) of these unusual unsubstituted ketene hemiaminals.

Pyridinium ketene hemiaminals are an emerging class of reagents in organic synthesis that are able to engage in a variety of reaction modes such as electrophilic aromatic substitutions, nucleophilic aromatic substitutions (S_N_Ar), and amidations (Shapiro *et al.*, 2018 ▸; McConnell *et al.*, 2021 ▸). As part of our ongoing efforts to explore the use of these compounds in valuable synthetic applications, we have sought to employ 2-halopyridinium ketene hemiaminals as facile electrophiles in mild S_N_Ar reactions with amine nucleophiles, en route to the bioactive 2-amino­pyridine products such as **IV** (Fig. 1 ▸). During the course of this study, 2-amino­pyridium ketene hemiaminal **IV** yielded high-quality crystals. Given the scarcity of X-ray analyses on this compound class, we were compelled to investigate the X-ray structure of **IV** in depth. 
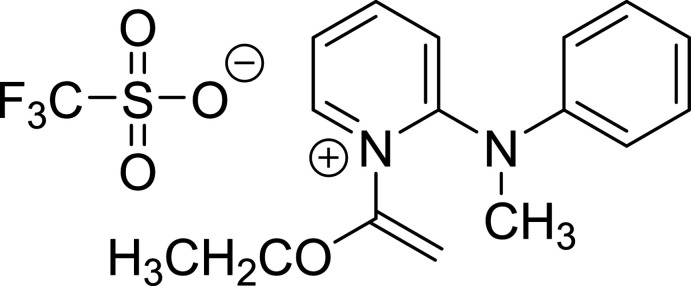


## 2. Structural commentary

**Figure 2:**
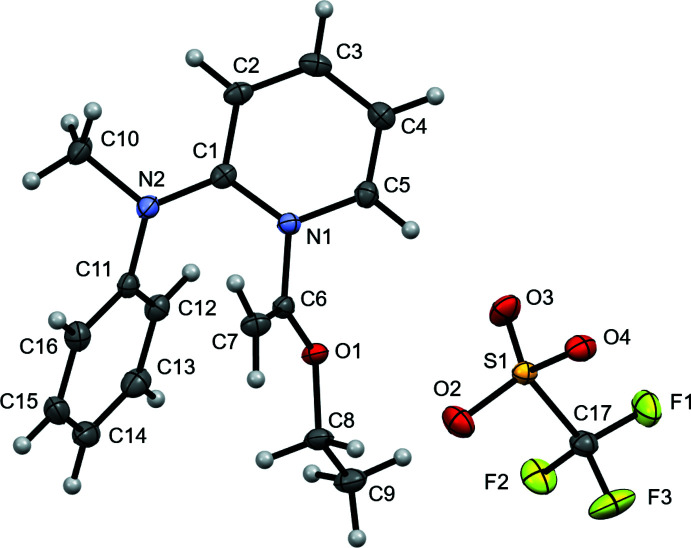
Structure and atomic numbering scheme of the title compound, shown as 50% probability ellipsoids.

The substituted pyridinium cation of the title compound is built from three individually planar fragments connected to form a non-coplanar mol­ecule (Fig. 2 ▸). The 2-(methyl­amino)­pyridine fragment forms one plane (*A*), the phenyl group extending from the amino-nitro­gen atom forms a second plane (*B*), and the eth­oxy­vinyl substituent extending from the pyridine-nitro­gen atom forms a third plane (*C*). Mean plane to mean plane angles between the fragments are 71.71 (4)° between *A* and *B*, 68.16 (4)° between *A* and *C*, and 29.77 (6)° between *B* and *C*. The phenyl group attached to the amino-nitro­gen atom is folded toward the same side of the amino­pyridine fragment as the eth­oxy­vinyl substituent, likely requiring their mean plane to mean plane angles to be closest to parallel. The orientation of the eth­oxy­vinyl substituent on the pyridine ring [C1—N1—C6—O1 torsion angle of 116.44 (12)°] is similar to that in the 2-chloro-substituted compound **I**, CSD refcode JETTOU, which has a mean plane to mean plane angle between the pyridine and eth­oxy­vinyl fragments of 70.2 (2)° and a C—N—C—O torsion angle of 109.1 (2)° about the exocyclic N—C bond, which was shown to be an energetically favorable arrangement (Shapiro *et al.*, 2018 ▸).

## 3. Supra­molecular features

**Figure 3:**
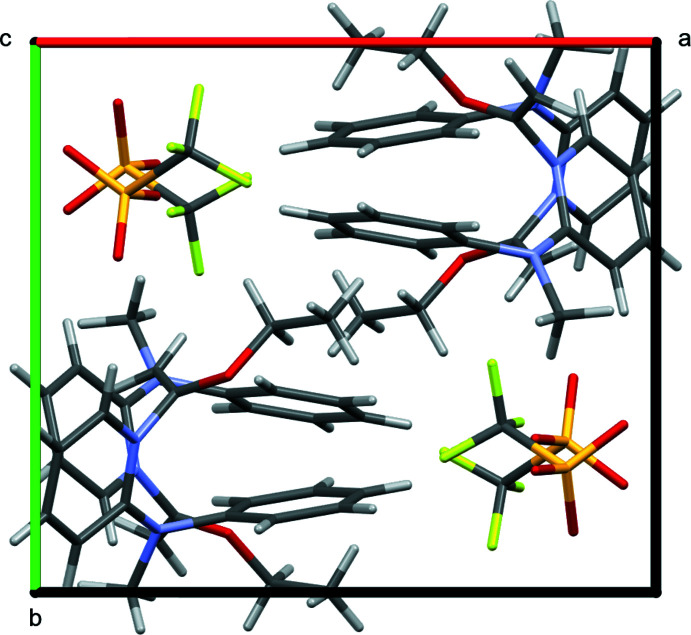
Packing of mol­ecules in the title compound, viewed along the *c*-axis.

**Figure 4:**
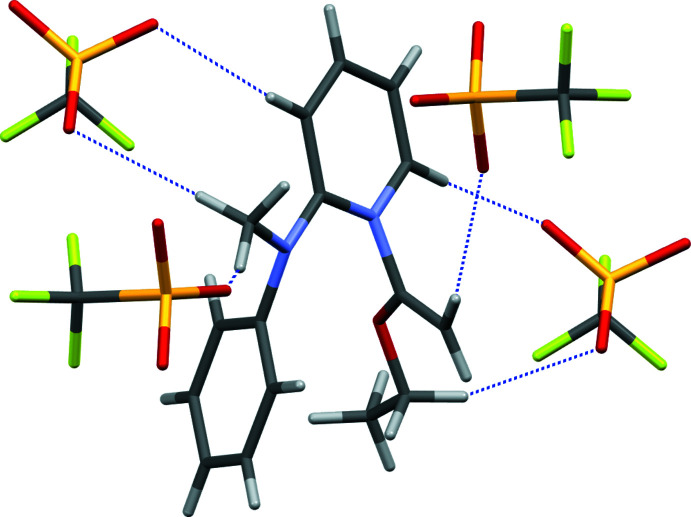
C—H⋯O inter­actions occurring between a central cation and four neighboring anions.

The triflate anions and substituted pyridinium cations are arranged in individual columns along the *c*-axis of the unit cell, and pack in alternating fashion along the *a*- and *b*-axes of the unit cell (Fig. 3 ▸). All three oxygen atoms of the triflate anion act as acceptor atoms for C—H⋯O inter­actions from the cation (Table 1 ▸). As a result, there are six C—H⋯O inter­actions between a central cation and four neighboring triflate anions where H⋯O is less than 2.60 Å (Fig. 4 ▸). The six contacts originate from the pyridinium fragment (two), methyl group on the amino nitro­gen atom (two), vinyl carbon atom (one), and eth­oxy group (one). The shortest contact occurs from C5 on the pyridinium ring, with H⋯O = 2.25 Å and C⋯O = 3.1819 (16) Å. Collectively, the six C—H⋯O inter­actions create a two-dimensional slab in the *bc* plane. These slabs may be considered to extend into a three-dimensional framework if a short C-H⋯F contact [H⋯F = 2.44 Å, C⋯F = 3.324 (2) Å, C—H⋯F = 153.1°] is considered from the C13 atom of the phenyl fragment to the F2 atom of the anion. Only one such contact occurs to the CF_3_ side of the anions.

## 4. Database survey

A CSD search revealed only six hits for any pyridinium-1-vinyl-1-ether fragment (CSD Version 5.43, Update 4, November 2022; Groom *et al.*, 2016 ▸). Of these, five were of substituted isoquinolinium salts, where the vinyl group of the searched fragment corresponds to a C=C bond in a thia­zole ring fused to the substituted iso­quinoline, making them largely unrelated to the title compound (Matsumoto *et al.*, 2018 ▸, 2022 ▸). The remaining hit is the related compound and precursor material, **I**, 2-chloro-1-(1-ethyoxyethen­yl)pyridin-1-ium tri­fluoro­methane­sulfonate, CSD refcode JETTOU (Shapiro *et al.*, 2018 ▸). Expansion of the search to include pyrazinium- or pyrimidinium-based fragments produced no hits.

## 5. Synthesis and crystallization

A sealed 0.5-2.0 mL Biotage microwave vial was charged with potassium carbonate (69 mg, 0.5 mmol), freshly prepared 2-chloro­pyridinium ketene hemiaminal **I** (167 mg, 0.5 mmol) (McConnell *et al.*, 2021 ▸) and di­chloro­methane (1 mL). While stirring the resulting suspension at room temperature, *N*-methyl­aniline (0.054 mL, 0.5 mmol) was slowly added. After one minute of stirring at room temperature, the sealed microwave vial was placed in a pre-heated 313 K oil bath and stirred for 24 h. The reaction mixture was cooled to room temperature then concentrated to a residue that was purified by silica gel column chromatography using a 0-70% gradient of iso­propanol in chloro­form to provide compound **IV** as a yellow solid (190 mg, 94%). ^1^H NMR (500 MHz, Acetone-*d*_6_) δ 8.36–8.26 (*m*, 2H), 7.69 (*d*, *J* = 9.1 Hz, 1H), 7.52 (*t*, *J* = 7.7 Hz, 2H), 7.45–7.38 (*m*, 4H), 4.69 (*d*, *J* = 4.8 Hz, 1H), 4.36 (*d*, *J* = 4.8 Hz, 1H), 3.74 (*s*, 3H), 3.63 (*q*, *J* = 7.1 Hz, 2H), 1.27 (*t*, *J* = 7.0 Hz, 3H); ^13^C NMR (125 MHz, CDCl_3_) δ 154.5, 152.6, 145.5, 144.2, 142.6, 129.8, 127.9, 126.4, 121.5 (*q*, ^1^ *J_C_*_F_ = 320 Hz, *C*F_3_), 119.5, 116.4, 85.9, 65.8, 43.4, 13.2.; LRMS–ES+ *m*/*z* (relative intensity) 255.1 (C_16_H_19_N_2_O *M*+, 100); HRMS–ES+ (C_16_H_19_N_2_O) calculated 255.1497 (*M*+), found 255.1497. X-ray quality crystals were formed by slow evaporation of a solution of the purified compound **IV** in acetone over the course of one week.

## 6. Refinement

Crystal data, data collection and structure refinement details are summarized in Table 2 ▸. Hydrogen atoms attached to carbon atoms were placed in calculated positions using appropriate riding models having C—H = 0.95–1.0 Å with *U*_iso_(H) = 1.5*U*_eq_(C) for methyl hydrogen atoms and *U*_iso_(H) = 1.2*U*_eq_(C) for other hydrogen atoms.

## Supplementary Material

Crystal structure: contains datablock(s) I. DOI: 10.1107/S2056989023005741/zl5048sup1.cif

Structure factors: contains datablock(s) I. DOI: 10.1107/S2056989023005741/zl5048Isup2.hkl

Supporting information file. DOI: 10.1107/S2056989023005741/zl5048Isup3.cml

CCDC reference: 2278022

Additional supporting information:  crystallographic information; 3D view; checkCIF report

## Figures and Tables

**Table 1:** Hydrogen-bond geometry (Å, °)

*D*—H⋯*A*	*D*—H	H⋯*A*	*D*⋯*A*	*D*—H⋯*A*
C2—H2⋯O4^i^	0.95	2.43	3.2287 (16)	141
C5—H5⋯O3	0.95	2.25	3.1819 (16)	165
C7—H7*B*⋯O4^ii^	0.95	2.48	3.2724 (16)	140
C8—H8*B*⋯O2	0.99	2.55	3.5022 (17)	160
C10—H10*B*⋯O3^iii^	0.98	2.59	3.3633 (16)	136
C10—H10*C*⋯O2^i^	0.98	2.58	3.5408 (18)	168

Symmetry codes: (i) 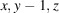 ; (ii) 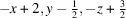 ; (iii) 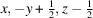 .

**Table 2:** Experimental details

Crystal data	
Chemical formula	C_16_H_19_N_2_O^+^·CF_3_O_3_S^−^
*M*_r_	404.40
Crystal system, space group	Monoclinic, *P*2_1_/*c*
Temperature (K)	100
*a*, *b*, *c* (Å)	13.0645 (11), 11.0190 (9), 13.4050 (11)
β (°)	107.826 (3)
*V* (Å^3^)	1837.1 (3)
*Z*	4
Radiation type	Cu *K*α
μ (mm^−1^)	2.08
Crystal size (mm)	0.19 × 0.14 × 0.11

Data collection	
Diffractometer	Bruker D8 Venture Photon 2
Absorption correction	Multi-scan (*SADABS*; Krause *et al.*, 2015 ▸)
*T*_min_, *T*_max_	0.779, 1.000
No. of measured, independent and observed [*I* > 2σ(*I*)] reflections	29494, 3598, 3389
*R*_int_	0.053
(sin θ/λ)_max_ (Å^−1^)	0.617

Refinement	
*R*[*F* ^2^ > 2σ(*F* ^2^)], *wR*(*F* ^2^), *S*	0.031, 0.080, 1.07
No. of reflections	3598
No. of parameters	246
H-atom treatment	H-atom parameters constrained
Δρ_max_, Δρ_min_ (e Å^−3^)	0.40, −0.38

Computer programs: *APEX3* (Bruker, 2017 ▸), *SAINT* (Bruker, 2016 ▸), *SHELXT2014/5* (Sheldrick, 2015*a* ▸), *SHELXL2016/6* (Sheldrick, 2015*b* ▸), *Mercury* (Macrae *et al.*, 2020 ▸), and *publCIF* (Westrip, 2010 ▸).

## supplementary crystallographic information

### Crystal data

**Table:**

C_16_H_19_N_2_O^+^·CF_3_O_3_S^−^	*F*(000) = 840
*M**_r_* = 404.40	*D*_x_ = 1.462 Mg m^−^^3^
Monoclinic, *P*2_1_/*c*	Cu *K*α radiation, λ = 1.54178 Å
*a* = 13.0645 (11) Å	Cell parameters from 9938 reflections
*b* = 11.0190 (9) Å	θ = 5.4–72.1°
*c* = 13.4050 (11) Å	µ = 2.08 mm^−^^1^
β = 107.826 (3)°	*T* = 100 K
*V* = 1837.1 (3) Å^3^	Block, yellow
*Z* = 4	0.19 × 0.14 × 0.11 mm

### Data collection

**Table:**

Bruker D8 Venture Photon 2 diffractometer	3389 reflections with *I* > 2σ(*I*)
Radiation source: Incoatec IµS	*R*_int_ = 0.053
φ and ω scans	θ_max_ = 72.2°, θ_min_ = 5.3°
Absorption correction: multi-scan (SADABS; Krause *et al.*, 2015)	*h* = −15→16
*T*_min_ = 0.779, *T*_max_ = 1.000	*k* = −13→13
29494 measured reflections	*l* = −16→16
3598 independent reflections	

### Refinement

**Table:**

Refinement on *F*^2^	Primary atom site location: dual
Least-squares matrix: full	Secondary atom site location: difference Fourier map
*R*[*F*^2^ > 2σ(*F*^2^)] = 0.031	Hydrogen site location: inferred from neighbouring sites
*wR*(*F*^2^) = 0.080	H-atom parameters constrained
*S* = 1.07	*w* = 1/[σ^2^(*F*_o_^2^) + (0.0329*P*)^2^ + 0.8661*P*] where *P* = (*F*_o_^2^ + 2*F*_c_^2^)/3
3598 reflections	(Δ/σ)_max_ = 0.001
246 parameters	Δρ_max_ = 0.40 e Å^−^^3^
0 restraints	Δρ_min_ = −0.38 e Å^−^^3^

### Special details

**Table:**

Geometry. All esds (except the esd in the dihedral angle between two l.s. planes) are estimated using the full covariance matrix. The cell esds are taken into account individually in the estimation of esds in distances, angles and torsion angles; correlations between esds in cell parameters are only used when they are defined by crystal symmetry. An approximate (isotropic) treatment of cell esds is used for estimating esds involving l.s. planes.

### Fractional atomic coordinates and isotropic or equivalent isotropic displacement parameters (Å^2^)

**Table:**

	*x*	*y*	*z*	*U*_iso_*/*U*_eq_	
O1	0.68941 (7)	0.39328 (8)	0.68939 (7)	0.01770 (19)	
N1	0.83913 (8)	0.27687 (9)	0.73215 (8)	0.0151 (2)	
N2	0.80276 (8)	0.11322 (9)	0.61208 (8)	0.0174 (2)	
C1	0.85607 (10)	0.15964 (11)	0.70752 (10)	0.0159 (2)	
C2	0.93228 (10)	0.09169 (11)	0.78502 (10)	0.0190 (3)	
H2	0.948282	0.010822	0.770205	0.023*	
C3	0.98306 (10)	0.14072 (12)	0.88065 (10)	0.0210 (3)	
H3	1.033550	0.093353	0.931944	0.025*	
C4	0.96178 (10)	0.26022 (12)	0.90440 (10)	0.0203 (3)	
H4	0.996391	0.294247	0.971375	0.024*	
C5	0.89038 (10)	0.32568 (11)	0.82896 (10)	0.0179 (3)	
H5	0.875417	0.407076	0.843271	0.022*	
C6	0.77414 (10)	0.36295 (11)	0.65709 (10)	0.0159 (2)	
C7	0.80485 (10)	0.40244 (12)	0.57808 (10)	0.0207 (3)	
H7A	0.764019	0.462911	0.532491	0.025*	
H7B	0.867858	0.370459	0.566633	0.025*	
C8	0.61847 (10)	0.48421 (12)	0.62699 (10)	0.0209 (3)	
H8A	0.591484	0.458094	0.552862	0.025*	
H8B	0.657063	0.562177	0.630412	0.025*	
C9	0.52670 (11)	0.49849 (13)	0.67165 (11)	0.0262 (3)	
H9A	0.486532	0.422138	0.663766	0.039*	
H9B	0.478850	0.563254	0.634162	0.039*	
H9C	0.554908	0.519366	0.746135	0.039*	
C10	0.84661 (11)	0.00327 (12)	0.57730 (11)	0.0230 (3)	
H10A	0.924596	0.011775	0.592838	0.034*	
H10B	0.813410	−0.007354	0.501672	0.034*	
H10C	0.830934	−0.067632	0.614292	0.034*	
C11	0.69110 (10)	0.13846 (11)	0.55991 (10)	0.0171 (3)	
C12	0.61681 (10)	0.13292 (11)	0.61476 (10)	0.0198 (3)	
H12	0.639600	0.114576	0.687501	0.024*	
C13	0.50860 (11)	0.15441 (12)	0.56254 (12)	0.0249 (3)	
H13	0.457657	0.151371	0.600081	0.030*	
C14	0.47469 (11)	0.18013 (12)	0.45653 (12)	0.0268 (3)	
H14	0.400837	0.195192	0.421303	0.032*	
C15	0.54952 (12)	0.18379 (13)	0.40186 (11)	0.0265 (3)	
H15	0.526424	0.200652	0.328851	0.032*	
C16	0.65735 (11)	0.16306 (12)	0.45289 (10)	0.0226 (3)	
H16	0.708102	0.165609	0.415102	0.027*	
C17	0.75323 (10)	0.80283 (12)	0.84257 (11)	0.0215 (3)	
S1	0.85507 (2)	0.72568 (3)	0.79769 (2)	0.01600 (10)	
F1	0.78050 (7)	0.80925 (10)	0.94640 (7)	0.0377 (2)	
F2	0.65920 (6)	0.74488 (9)	0.80939 (7)	0.0324 (2)	
F3	0.73626 (7)	0.91547 (8)	0.80509 (9)	0.0408 (2)	
O2	0.80593 (8)	0.72136 (10)	0.68618 (7)	0.0281 (2)	
O3	0.86690 (8)	0.61097 (8)	0.85145 (8)	0.0267 (2)	
O4	0.94731 (7)	0.80419 (9)	0.83385 (8)	0.0241 (2)	

### Atomic displacement parameters (Å^2^)

**Table:**

	*U*^11^	*U*^22^	*U*^33^	*U*^12^	*U*^13^	*U*^23^
O1	0.0169 (4)	0.0165 (4)	0.0194 (4)	0.0043 (3)	0.0050 (3)	0.0024 (3)
N1	0.0145 (5)	0.0137 (5)	0.0163 (5)	0.0008 (4)	0.0038 (4)	0.0012 (4)
N2	0.0163 (5)	0.0155 (5)	0.0203 (5)	0.0011 (4)	0.0055 (4)	−0.0028 (4)
C1	0.0152 (6)	0.0145 (6)	0.0198 (6)	−0.0007 (4)	0.0079 (5)	0.0009 (5)
C2	0.0178 (6)	0.0148 (6)	0.0252 (6)	0.0013 (5)	0.0079 (5)	0.0036 (5)
C3	0.0164 (6)	0.0221 (6)	0.0230 (6)	0.0010 (5)	0.0039 (5)	0.0074 (5)
C4	0.0188 (6)	0.0231 (6)	0.0179 (6)	−0.0017 (5)	0.0039 (5)	0.0003 (5)
C5	0.0176 (6)	0.0172 (6)	0.0192 (6)	−0.0018 (5)	0.0060 (5)	−0.0016 (5)
C6	0.0156 (6)	0.0119 (5)	0.0186 (6)	0.0002 (4)	0.0026 (5)	−0.0009 (4)
C7	0.0206 (6)	0.0181 (6)	0.0233 (6)	0.0023 (5)	0.0068 (5)	0.0039 (5)
C8	0.0199 (6)	0.0182 (6)	0.0226 (6)	0.0059 (5)	0.0037 (5)	0.0045 (5)
C9	0.0232 (7)	0.0262 (7)	0.0288 (7)	0.0090 (5)	0.0074 (6)	0.0037 (6)
C10	0.0251 (7)	0.0174 (6)	0.0277 (7)	0.0030 (5)	0.0100 (5)	−0.0048 (5)
C11	0.0177 (6)	0.0126 (6)	0.0200 (6)	−0.0014 (4)	0.0042 (5)	−0.0028 (4)
C12	0.0212 (6)	0.0169 (6)	0.0215 (6)	−0.0006 (5)	0.0072 (5)	−0.0008 (5)
C13	0.0207 (6)	0.0211 (6)	0.0343 (8)	−0.0011 (5)	0.0104 (6)	−0.0035 (6)
C14	0.0194 (6)	0.0201 (7)	0.0344 (8)	0.0009 (5)	−0.0013 (6)	−0.0044 (6)
C15	0.0309 (7)	0.0230 (7)	0.0199 (6)	0.0008 (6)	−0.0006 (6)	−0.0025 (5)
C16	0.0252 (7)	0.0225 (6)	0.0199 (6)	−0.0018 (5)	0.0069 (5)	−0.0022 (5)
C17	0.0172 (6)	0.0229 (6)	0.0234 (6)	0.0002 (5)	0.0044 (5)	0.0002 (5)
S1	0.01580 (16)	0.01503 (17)	0.01710 (17)	−0.00019 (10)	0.00493 (12)	0.00136 (10)
F1	0.0340 (5)	0.0542 (6)	0.0244 (4)	0.0069 (4)	0.0084 (4)	−0.0126 (4)
F2	0.0182 (4)	0.0450 (5)	0.0356 (5)	−0.0072 (4)	0.0107 (3)	−0.0031 (4)
F3	0.0333 (5)	0.0251 (5)	0.0691 (7)	0.0124 (4)	0.0229 (5)	0.0117 (4)
O2	0.0244 (5)	0.0408 (6)	0.0189 (5)	−0.0010 (4)	0.0065 (4)	0.0007 (4)
O3	0.0359 (5)	0.0162 (5)	0.0306 (5)	0.0019 (4)	0.0141 (4)	0.0038 (4)
O4	0.0159 (4)	0.0209 (5)	0.0340 (5)	−0.0014 (4)	0.0056 (4)	0.0006 (4)

### Geometric parameters (Å, º)

**Table:**

O1—C6	1.3484 (15)	C9—H9B	0.9800
O1—C8	1.4450 (14)	C9—H9C	0.9800
N1—C1	1.3679 (16)	C10—H10A	0.9800
N1—C5	1.3746 (16)	C10—H10B	0.9800
N1—C6	1.4526 (15)	C10—H10C	0.9800
N2—C1	1.3558 (16)	C11—C12	1.3869 (18)
N2—C11	1.4387 (16)	C11—C16	1.3924 (18)
N2—C10	1.4754 (16)	C12—C13	1.3923 (19)
C1—C2	1.4135 (17)	C12—H12	0.9500
C2—C3	1.3619 (19)	C13—C14	1.382 (2)
C2—H2	0.9500	C13—H13	0.9500
C3—C4	1.4024 (19)	C14—C15	1.390 (2)
C3—H3	0.9500	C14—H14	0.9500
C4—C5	1.3548 (18)	C15—C16	1.384 (2)
C4—H4	0.9500	C15—H15	0.9500
C5—H5	0.9500	C16—H16	0.9500
C6—C7	1.3161 (18)	C17—F1	1.3288 (16)
C7—H7A	0.9500	C17—F3	1.3319 (16)
C7—H7B	0.9500	C17—F2	1.3342 (16)
C8—C9	1.5033 (19)	C17—S1	1.8289 (14)
C8—H8A	0.9900	S1—O2	1.4357 (10)
C8—H8B	0.9900	S1—O3	1.4397 (10)
C9—H9A	0.9800	S1—O4	1.4415 (10)
			
C6—O1—C8	115.47 (9)	H9A—C9—H9C	109.5
C1—N1—C5	121.95 (10)	H9B—C9—H9C	109.5
C1—N1—C6	123.57 (10)	N2—C10—H10A	109.5
C5—N1—C6	114.26 (10)	N2—C10—H10B	109.5
C1—N2—C11	122.50 (10)	H10A—C10—H10B	109.5
C1—N2—C10	118.10 (10)	N2—C10—H10C	109.5
C11—N2—C10	116.05 (10)	H10A—C10—H10C	109.5
N2—C1—N1	120.64 (11)	H10B—C10—H10C	109.5
N2—C1—C2	122.44 (11)	C12—C11—C16	120.17 (12)
N1—C1—C2	116.90 (11)	C12—C11—N2	120.25 (11)
C3—C2—C1	120.81 (12)	C16—C11—N2	119.51 (11)
C3—C2—H2	119.6	C11—C12—C13	119.57 (12)
C1—C2—H2	119.6	C11—C12—H12	120.2
C2—C3—C4	120.85 (12)	C13—C12—H12	120.2
C2—C3—H3	119.6	C14—C13—C12	120.55 (13)
C4—C3—H3	119.6	C14—C13—H13	119.7
C5—C4—C3	118.00 (12)	C12—C13—H13	119.7
C5—C4—H4	121.0	C13—C14—C15	119.47 (13)
C3—C4—H4	121.0	C13—C14—H14	120.3
C4—C5—N1	121.43 (12)	C15—C14—H14	120.3
C4—C5—H5	119.3	C16—C15—C14	120.55 (13)
N1—C5—H5	119.3	C16—C15—H15	119.7
C7—C6—O1	131.23 (11)	C14—C15—H15	119.7
C7—C6—N1	121.05 (11)	C15—C16—C11	119.66 (13)
O1—C6—N1	107.61 (10)	C15—C16—H16	120.2
C6—C7—H7A	120.0	C11—C16—H16	120.2
C6—C7—H7B	120.0	F1—C17—F3	107.80 (11)
H7A—C7—H7B	120.0	F1—C17—F2	107.39 (11)
O1—C8—C9	106.88 (10)	F3—C17—F2	106.88 (11)
O1—C8—H8A	110.3	F1—C17—S1	112.37 (9)
C9—C8—H8A	110.3	F3—C17—S1	111.29 (9)
O1—C8—H8B	110.3	F2—C17—S1	110.87 (9)
C9—C8—H8B	110.3	O2—S1—O3	115.94 (6)
H8A—C8—H8B	108.6	O2—S1—O4	115.55 (6)
C8—C9—H9A	109.5	O3—S1—O4	114.08 (6)
C8—C9—H9B	109.5	O2—S1—C17	102.67 (6)
H9A—C9—H9B	109.5	O3—S1—C17	102.76 (6)
C8—C9—H9C	109.5	O4—S1—C17	103.17 (6)
			
C11—N2—C1—N1	−40.23 (17)	C1—N2—C11—C12	−45.16 (17)
C10—N2—C1—N1	161.27 (11)	C10—N2—C11—C12	113.75 (13)
C11—N2—C1—C2	140.95 (12)	C1—N2—C11—C16	137.76 (13)
C10—N2—C1—C2	−17.55 (17)	C10—N2—C11—C16	−63.34 (15)
C5—N1—C1—N2	178.69 (11)	C16—C11—C12—C13	−1.23 (19)
C6—N1—C1—N2	−6.97 (17)	N2—C11—C12—C13	−178.29 (11)
C5—N1—C1—C2	−2.43 (17)	C11—C12—C13—C14	0.6 (2)
C6—N1—C1—C2	171.92 (11)	C12—C13—C14—C15	0.4 (2)
N2—C1—C2—C3	−178.96 (12)	C13—C14—C15—C16	−0.6 (2)
N1—C1—C2—C3	2.18 (18)	C14—C15—C16—C11	−0.1 (2)
C1—C2—C3—C4	−0.58 (19)	C12—C11—C16—C15	0.98 (19)
C2—C3—C4—C5	−0.85 (19)	N2—C11—C16—C15	178.07 (12)
C3—C4—C5—N1	0.63 (19)	F1—C17—S1—O2	175.89 (10)
C1—N1—C5—C4	1.07 (18)	F3—C17—S1—O2	−63.11 (11)
C6—N1—C5—C4	−173.76 (12)	F2—C17—S1—O2	55.70 (11)
C8—O1—C6—C7	0.53 (19)	F1—C17—S1—O3	55.19 (11)
C8—O1—C6—N1	176.61 (9)	F3—C17—S1—O3	176.19 (9)
C1—N1—C6—C7	−67.00 (16)	F2—C17—S1—O3	−65.00 (10)
C5—N1—C6—C7	107.74 (14)	F1—C17—S1—O4	−63.67 (11)
C1—N1—C6—O1	116.44 (12)	F3—C17—S1—O4	57.33 (11)
C5—N1—C6—O1	−68.82 (12)	F2—C17—S1—O4	176.15 (9)
C6—O1—C8—C9	176.38 (10)		

### Hydrogen-bond geometry (Å, º)

**Table:**

*D*—H···*A*	*D*—H	H···*A*	*D*···*A*	*D*—H···*A*
C2—H2···O4^i^	0.95	2.43	3.2287 (16)	141
C5—H5···O3	0.95	2.25	3.1819 (16)	165
C7—H7*B*···O4^ii^	0.95	2.48	3.2724 (16)	140
C8—H8*B*···O2	0.99	2.55	3.5022 (17)	160
C10—H10*B*···O3^iii^	0.98	2.59	3.3633 (16)	136
C10—H10*C*···O2^i^	0.98	2.58	3.5408 (18)	168

Symmetry codes: (i) *x*, *y*−1, *z*; (ii) −*x*+2, *y*−1/2, −*z*+3/2; (iii) *x*, −*y*+1/2, *z*−1/2.
